# Multi-Frequency Based Direction-of-Arrival Estimation for *2q*-Level Nested Radar & Sonar Arrays

**DOI:** 10.3390/s18103385

**Published:** 2018-10-10

**Authors:** Hao Zhou, Guoping Hu, Junpeng Shi, Ziang Feng

**Affiliations:** 1 Air and Missile Defense College, Air Force Engineering University, Xi’an 710051, China; 17792611529@126.com (H.Z.); netbigangang@163.com (Z.F.); 2 Electronic Countermeasures College, National University of Defense Technology, Hefei 230000, China; 15667081720@163.com

**Keywords:** radar, sonar, 2*q*th-order cumulant, difference co-array, degree of freedom (DOF), multi-frequency, nested array

## Abstract

Direction finding is a hot research area in radar and sonar systems. In the case of *q* ≥ 2, the 2*q*th-order cumulant based direction of arrival (DOA) estimation algorithm for the *2q*-level nested array can achieve high resolution performance. A virtual *2q*th-order difference co-array, which contains *O*(*N*^2*q*^) virtual sensors in the form of a uniform linear array (ULA), is yielded and the Gaussian noise is eliminated. However, some virtual elements are separated by the holes among the *2q*th-order difference co-array and cannot be fully used. Even though the application of the multi-frequency method for minimum frequency separation (MFMFS) can fill the holes with low computation complexity, it requires that the number of frequencies must increase with the number of holes. In addition, the signal spectra have to be proportional for all frequencies, which is hard to satisfy when the number of holes is large. Aiming at this, we further propose a multi-frequency method for a minimum number of frequencies (MFMNF) and discuss the best frequency choice under two specific situations. Simulation results verify that, compared with the MFMFS method, the proposed MFMNF method can use only one frequency to fill all the holes while achieving a longer virtual array and the DOA estimation performance is, therefore, improved.

## 1. Introduction

Non-uniform arrays have been widely used in the radar and sonar systems. They have many advantages over traditional uniform arrays including higher resolution and less redundancy [1,2]. Though the minimum redundancy arrays (MRA) [3] and minimum hole arrays (MHA) [4] have been proposed decades ago, they are not widely used in practice for the lack of closed-form array configuration expressions, which means an exhaustive search through all possible combinations of the sensors are required to find the optimal design. As a special kind of non-uniform array, the random arrays have lower computation complexity and satisfactory resolution compared with the traditional uniform linear arrays (ULA). Compressive sensing (CS) algorithms such as the orthogonal matching pursuit (OMP), the basis pursuit (BP), and the multi-branch matching pursuit (MBMP) are applied in the random array for a direction-of-arrival (DOA) estimation [5,6,7]. Different from the random array whose sensors’ positions are random, the nested array [8] and co-prime array [9] have closed-form expressions of sensors’ positions and their degrees of freedom (DOFs) are expanded. Due to the flexible configuration and diversity characteristic, the multi-input multi-output (MIMO) structure is combined with the nested or co-prime array to obtain bigger DOFs and higher resolution. Shi et al. utilize a co-prime pair of ULA within the MIMO radar framework [10] and achieve a better DOA estimation performance. However, since only the sum co-array is employed in the proposed array, the provided DOFs are limited. In Reference [11,12], the difference co-array of the sum co-array (DCSC) is taken into consideration and further increases the DOFs. Except for the optimization of physical array geometry, classical DOA estimation algorithms are combined with new techniques to improve the DOA estimation performance. For example, References [13,14] combine the time reversal (TR) with the multiple signal classification (MUSIC) and maximum likelihood (ML) algorithm and the DOA estimation performance are effectively improved. References [15,16] further study the performance of TR-MUSIC and derived the closed form of the mean-squared error (MSE) matrix of TR-MUSIC. These approaches are based on the second-order statistics of the data. Since the 2*q*th-order cumulant of Gaussian noise is zero when q≥2, the negative effect of noise will be degraded and the DOA estimation performance based on the 2*q*th-order cumulant will be improved [17,18]. The *2q*th-order cumulant can yield an equivalent higher order virtual array with larger DOFs, which improves the DOA estimation performance [19]. To fully utilize the DOFs provided by the *2q*th-order cumulant-based algorithms, the geometry of the physical array has to be optimized correspondingly. Therefore, Pal et al. propose the *2q*-level nested array in which the 2*q*th-order difference co-array yields a virtual ULA with $O\left(N^{2q}\right)$ elements and can identify up to $O\left(N^{2q}\right)$ sources with only O(N) sensors [20]. This kind of array can be applied to the practical weather radar where higher-order moments are required to retrieve the information [21]. However, apart from the hole-free 2-level nested array, the *2q*-level nested array has holes in the *2q*th-order difference co-array. Therefore, some discrete virtual sensors cannot be fully utilized for the subspace based DOA estimation algorithms such as MUSIC, estimation of signal parameters via rotational invariance techniques (ESPRIT), etc. Some studies only use the continuous elements in the difference co-array, but the discrete elements are wasted [22]. Aiming at this problem, Y. Iizuka et al. propose an optimal linear array with minimum redundancy (OLA-MR) based on the 2*q*th-order cumulant [23]. The proposed array maximizes the continuous DOFs by simulated annealing. Matrix completion processing is another approach to solve the problem by filling the holes in the *2q*-level nested array to exploit all the provided DOFs [24,25]. However, both the OLA-MR method and the matrix completion processing are not efficient in practical applications because, with the increase of the number of sensors, the optimization will be more complicated and time consuming. As a new approach, multi-frequency operation [26] has been applied in DOA estimation. Reference [27] utilizes multi-frequencies for scene probing and proposes time-reversal based imaging algorithms in wideband sources. Moulton [28] and BouDaher [29] utilize multiple frequencies to fill the missing co-array elements in order for all of the offered DOFs to be effectively utilized.

Inspired by the above research, we first apply the multi-frequency method to fill the holes in the *2q*th-order difference co-array. Additionally, the isolated virtual elements can be connected to yield a longer continuous ULA. This method is the optimal from the aspect of frequency separation. However, the number of required frequencies equals half of the number of holes. A lot of frequencies will be needed when the number of holes is large. The non-proportional signal spectra problem will become serious when many frequencies are used. Aiming at these disadvantages, we further propose the optimized multi-frequency method from the aspect of using a minimum number of frequencies. For two specific situations, we give different solutions and both of them can fill the holes while extending the DOFs of the continuous ULA with only one additional frequency. At the same time, the requirement of proportional signal spectra is easier to satisfy. The virtual DOFs can be fully utilized and DOA estimation performance can be improved. 

The remainder of the paper is organized as follows. In Section 2, the *2q*th-order cumulant and the *2q*th-order difference co-array are introduced. Next, the model of the *2q*-level nested array is constructed. In Section 3, the multi-frequency method for minimum frequency separation (MFMFS) is introduced. Aiming at the disadvantages of the MFMFS method, we further propose the multi-frequency method for a minimum number of frequencies (MFMNF). In addition, the best frequency choices under two specific situations are discussed. Several simulations are presented in Section 4 to validate the proposed methods. Lastly, Section 5 concludes the paper. Throughout the paper, ${\left[\cdot \right]}^{T}$, ${\left[\cdot \right]}^{*}$ and ${\left[\cdot \right]}^{H}$ denote the transpose, the conjugate, and the conjugate transpose of the matrix, respectively. ⊗ denotes the Kronecker product and ${\left[\cdot \right]}_{i,j}$ denotes the (i,j)th element of the matrix.

## 2. 2*q*th-Order Cumulant, 2*q*th-Order Difference Co-Array and 2*q*-Level Nested Array

### 2.1. 2qth-Order Cumulant Matrix

In recent years, the *2q*th-order cumulant has been applied in DOA estimation. Suppose *D* independent sources impinging on a linear array with N omni-directional sensors. The received signal vector of the N sensors is shown below.

(1) $$y\left(t\right)=\sum_{d=1}^{D}a\left(\theta_{d}\right)s_{d}\left(t\right)+u\left(t\right),$$

Since the 2*q*th-order cumulants of Gaussian sources are zero when q≥2, the source signals are assumed to be binary phase-shift keying (BPSK) modulated, which are non-Gaussian distributed. Therefore, the source signal can be modeled as $s_{d}\left(t\right)=\sigma_{d}e^{i\phi_{d}}\varepsilon \left(t\right)$ (d=1,2,⋯,D; t=1,2,⋯P) where ε(t) takes value ±1 with equal probability. P is the number of snapshots. $\sigma_{d}$ and $\phi_{d}$ denote the amplitude and phase of the signal, respectively, and they are considered as unknown parameters. u(t) is the N×1 zero mean stationary Gaussian noise vector. $a\left(\theta_{d}\right)=\left[1,e^{j\frac{2\pi }{\lambda }x_{1}\sin\theta_{d}},\cdots ,e^{j\frac{2\pi }{\lambda }x_{N-1}\sin\theta_{d}}\right]$ is the N×1 corresponding steering vector of the *d*th source in which $x_{n}$ (n=0,1,⋯,N−1) denotes the distance from the *n*th sensor to the reference sensor. $y\left(t\right)={\left[y_{1}\left(t\right),y_{2}\left(t\right),\cdots ,y_{N}\left(t\right)\right]}^{T}$. By calculating the 2*q*th-order cumulants of the elements in y(t) and arranging them, we can obtain an $N^{q}\times N^{q}$ *2q*th-order Hermitian matrix. According to Reference [20], when q≥2, there are more than one arrangement form of the cumulant matrix. Using $C_{2q,y}\left(l\right)$ (0≤l≤q−1) to denote different types of cumulant matrices, the equation below is developed.

(2) $$C_{2q,y}\left(l\right)=\sum_{d=1}^{D}c_{2q,s_{d}}\left[a{\left(\theta_{d}\right)}^{⊗l}⊗a{\left(\theta_{d}\right)}^{*⊗\left(q-l\right)}\right]\times {\left[a{\left(\theta_{d}\right)}^{⊗l}⊗a{\left(\theta_{d}\right)}^{*⊗\left(q-l\right)}\right]}^{H}+\sigma_{u}^{2}I_{N^{q}\times N^{q}}\delta \left(q-1\right),$$

where $c_{2q,s_{d}}=\mathrm{Cum}\left[s_{d_{1}}\left(t\right),\cdots ,s_{d_{q}}\left(t\right),s_{d_{q+1}}^{*}\left(t\right),\cdots ,s_{d_{2q}}^{*}\left(t\right)\right]$ with $d_{j}=d,1\leq j\leq 2q$. Cum[⋅] denotes the 2*q*th-order cumulant of the elements [20]. $a{\left(\theta_{d}\right)}^{⊗l}$ is the $N^{l}\times 1$ vector defined by the equation below.

(3) $$a{\left(\theta_{d}\right)}^{⊗l}≜\underset{\begin{array}{cc} l & times \end{array}}{\underset{︸}{a\left(\theta_{d}\right)⊗a\left(\theta_{d}\right)⊗\cdots ⊗a\left(\theta_{d}\right)}}.$$

$\sigma_{u}^{2}$ is the noise power. When q≥2, the *2q*th-order cumulant for the Gaussian noise is zero. Therefore, the *2q*th-order cumulant is written as (2), where δ(⋅) is an impulsive function.

### 2.2. 2qth-Order Difference Co-Array

Vectorize the cumulant matrix in (2) into a column vector, i.e.,

(4) $$\begin{array}{ll} c_{\mathrm{vec}}\left(l\right) & =\mathrm{vec}\left[C_{2q,y}\left(l\right)\right]\\ & =\mathrm{vec}\left[\sum_{d=1}^{D}\xi_{2q,s_{d}}a_{\mathrm{virtual}}^{\left(l\right)}\left(\theta_{d}\right)a_{\mathrm{virtual}}^{\left(l\right)}{\left(\theta_{d}\right)}^{H}\right]\\ & =\sum_{d=1}^{D}\xi_{2q,s_{d}}a_{\mathrm{virtual}}^{2q}\left(\theta_{d}\right)\\ & =B_{2q}\left(\theta \right)\eta \end{array}$$

where $B_{2q}\left(\theta \right)=\left[a_{\mathrm{virtual}}^{2q}\left(\theta_{1}\right),a_{\mathrm{virtual}}^{2q}\left(\theta_{2}\right),\cdots ,a_{\mathrm{virtual}}^{2q}\left(\theta_{D}\right)\right]\in {\mathbb{C}}^{N^{2q}\times D}$ ($\theta ={\left[\theta_{1},\theta_{2},\cdots ,\theta_{D}\right]}^{T}$) denotes the manifold of the virtual *2q*th-order difference co-array after vectorization and $\eta \in {\mathbb{C}}^{D\times 1}$ is the vector consisted by the *2q*th-order cumulants of the sources.

(5) $$a_{\mathrm{virtual}}^{2q}\left(\theta_{d}\right)=a_{\mathrm{virtual}}^{\left(l\right)*}\left(\theta_{d}\right)⊗a_{\mathrm{virtual}}^{\left(l\right)}\left(\theta_{d}\right).$$

The elements in $a_{\mathrm{virtual}}^{2q}\left(\theta_{d}\right)$ are expressed by the formula below.

(6) $$\begin{array}{l} e^{j\frac{2\pi }{\lambda }\left(\sum_{i=1}^{l}x_{n_{i}}-\sum_{k=l+1}^{q}x_{n_{k}}-\sum_{j=q+1}^{q+l}x_{n_{j}}+\sum_{m=q+l+1}^{2q}x_{n_{m}}\right)\sin\theta_{d}}\\ =e^{j\frac{2\pi }{\lambda }\left(\sum_{i=1}^{l}z_{i}-\sum_{k=q+1}^{2q}z_{k}\right)\sin\theta_{d}} \end{array}$$

where

(7) $$z_{i}=\{\begin{array}{l} x_{n_{i}}\;1\leq i\leq l,\;q+1\leq i\leq q+l\\ x_{n_{i+q}}\;l+1\leq i\leq q\\ x_{n_{i-q}}\;q+l+1\leq i\leq 2q \end{array}.\;$$

The set

(8) $$\left\{\sum_{i=1}^{q}z_{i}-\sum_{k=q+1}^{2q}z_{k},\;z_{i},z_{k}\in \left\{x_{n},\;n=1,2,\cdots ,N\right\}\right\}\;$$

is the sensors position set of the *2q*th-order difference co-array. Regardless of different values of l, the *2q*th-order difference co-array of the physical array is the same.

### 2.3. 2q-Level Nested Array

The *2q*-level nested array is a non-uniform array consisting of *2q* ULAs and the number of physical sensors is $N=\sum_{i=1}^{2q}\left(N_{i}-1\right)+1$.

For a given number of physical sensors, the optimal numbers of each level ULA are shown below.

(9) $$\begin{array}{l} N_{i}=m+1,\;i=1,2,\cdots ,n\\ N_{i}=m,\;\;\;i=n+1,n+2,\cdots ,2q \end{array}$$

where m and n are the quotient and remainder of N+2q−1 modulo 2q.

When 1≤i≤2q−1, the *i*th ULA contains $N_{i}-1$ sensors and their positions are given by the formula below.

(10) $$\left\{n\left(\prod_{k=0}^{i-1}N_{k}\right)\lambda /2,n=1,2,\cdots ,N_{i}-1\right\}.$$

The *2q* ULA contains $N_{2q}$ sensors located at:

(11) $$\left\{n\left(\prod_{k=0}^{2q-1}N_{k}\right)\lambda /2,n=1,2,\cdots ,N_{2q}\right\}.$$

**Figure 1:**
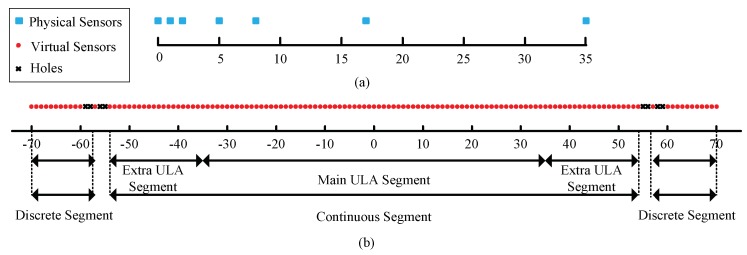
Example of the four-level nested array with *N* = 7 physical sensors. (**a**) Sensors positions of the physical array (*N* = 7). (**b**) Corresponding fourth-order difference co-array.

To demonstrate the structure of the *2q*-level nested array more clearly, suppose a four-level nested array (*q* = 2) with *N* = 7 sensors. The numbers of sensors from the first to fourth level ULA are 2, 2, 1, and 2, respectively. Suppose the first sensor is located at 0, then the positions of these seven physical sensors are [0,1,2,5,8,17,35]. Figure 1 shows the four-level nested array and the corresponding fourth-order difference co-array. It can be observed from Figure 1 that the main ULA segment of the fourth-order difference co-array is from −35 to 35 and the extra ULA segment on the positive side extends from 36 to 54 and the negative side extends from −36 to −54. Therefore, the discrete segment has 12 elements apart from the continuous segment on each side. In a previous paper [20], only the continuous segment is utilized in the DOA estimation algorithm, which actually wastes some DOFs. Inspired by the method discussed in Reference [28,29], multi-frequency methods for a 2*q*-level nested array are proposed in the following section.

## 3. Multi-Frequency Method and Its Application in a 2*q*-Level Nested Array

To fill the holes in the 2*q*th-order difference co-array, we propose two kinds of multi-frequency methods under different optimization requirements in this section.

### 3.1. The Multi-Frequency Method for Minimum Frequency Seperation (MFMFS)

First, inspired by the multi-frequency method in References [28,29], a direct idea is to let sensors next to the holes operate at multi-frequencies to yield equivalent virtual elements located at the positions of holes. This method is optimal from the aspect of minimum frequency separation.

Assume that the former (1) is working at frequency $\omega_{0}$, then (4) can be presented in another form.

(12) $$c_{\mathrm{vec}}\left(\omega_{0}\right)=B_{2q}\left(\omega_{0}\right)\eta \left(\omega_{0}\right).$$

Average the repetitive elements in $c_{\mathrm{vec}}\left(\omega_{0}\right)$ and then rank them in the ascension order of sensors positions to yield ${\overset{⌢}{c}}_{\mathrm{vec}}\left(\omega_{0}\right)$. Define a $N^{2q\times 1}$ support vector $p\left(\omega_{0}\right)$, among which the formula is below.

(13) $${\left[p\left(\omega_{0}\right)\right]}_{N^{q}\left(j-1\right)+i,1}=\Xi \left\{{\left[B_{2q}\left(\omega_{0}\right)\right]}_{i,j}\right\}\left(1\leq i,j\leq N^{q}\right),$$

where ${\left[p\left(\omega_{0}\right)\right]}_{N^{q}\left(j-1\right)+i,1}$ is the element in the $N^{q}\left(j-1\right)+i,1$th row of $p\left(\omega_{0}\right)$ and ${\left[B_{2q}\left(\omega_{0}\right)\right]}_{i,j}$ is the element in *i*th row and *j*th column of $B_{2q}\left(\omega_{0}\right)$. $\Xi \left\{{\left[B_{2q}\left(\omega_{0}\right)\right]}_{i,j}\right\}$ denotes the sensor position of ${\left[B_{2q}\left(\omega_{0}\right)\right]}_{i,j}$. Similarly, after extracting the unique elements in $p\left(\omega_{0}\right)$ and ranking them in an ascending order, we get $\overset{⌢}{p}\left(\omega_{0}\right)$, which actually represents the positions of elements in ${\overset{⌢}{c}}_{\mathrm{vec}}\left(\omega_{0}\right)$. By analyzing $\overset{⌢}{p}\left(\omega_{0}\right)$, the positions of holes (denoted as $\gamma_{\mathrm{hole}}$) are obtained. 

Now, let the physical array operate at another frequency $\omega_{h}=\alpha_{h}\omega_{0}$, i.e., [29]

(14) $$y\left(\omega_{h}\right)=A\left(\omega_{h}\right)s\left(\omega_{h}\right)+u\left(\omega_{h}\right),$$

where $\alpha_{h}=\gamma_{\mathrm{hole}}\left(h\right)/\gamma_{\mathrm{side}}\left(h\right)$(h=1,2,⋯,H). H is the total number of holes and $\gamma_{\mathrm{side}}\left(h\right)$ is the sensor position next to the hth hole. $A\left(\omega_{h}\right)$ is the physical array manifold and its (i,j)th element can be expressed by the equation below.

(15) $${\left[A\left(\omega_{h}\right)\right]}_{i,j}=e^{jk_{h}x_{i}\sin\left(\theta_{j}\right)}=e^{j\alpha_{h}k_{0}x_{i}\sin\left(\theta_{j}\right)},$$

where $k_{h}=\omega_{h}/c=\alpha_{h}\omega_{0}/c=\alpha_{h}k_{0}$. Therefore, when $x_{i}=\gamma_{\mathrm{side}}\left(h\right)$, we create the equation below.

(16) $$\alpha_{h}x_{i}=\frac{\gamma_{\mathrm{hole}}\left(h\right)}{\gamma_{\mathrm{side}}\left(h\right)}\gamma_{\mathrm{side}}\left(h\right)=\gamma_{\mathrm{hole}}\left(h\right).$$

Equation (16) indicates that the element in $\gamma_{\mathrm{side}}\left(h\right)$ under frequency $\omega_{h}$ is equivalent to the element in $\gamma_{\mathrm{hole}}\left(h\right)$ under frequency $\omega_{0}$. 

Furthermore, Equation (4) can be rewritten by the formula below.

(17) $$c_{\mathrm{vec}}\left(\omega_{h}\right)=B_{2q}\left(\omega_{h}\right)\eta \left(\omega_{h}\right).$$

The revised support vector of $B_{2q}\left(\omega_{h}\right)$ can be extracted as $\overset{⌢}{p}\left(\omega_{h}\right)$. According to the above conclusion, the element in $\gamma_{\mathrm{side}}\left(h\right)$ of $\overset{⌢}{p}\left(\omega_{h}\right)$ happens to be the missing hole in $\gamma_{\mathrm{hole}}\left(h\right)$ of $\overset{⌢}{p}\left(\omega_{0}\right)$. Therefore, by extracting the corresponding elements in $\overset{⌢}{p}\left(\omega_{h}\right)$ (*h* = 1,2, …, *H*), which, in fact, equals the missing holes in $\overset{⌢}{p}\left(\omega_{0}\right)$ and fills the holes in $\overset{⌢}{p}\left(\omega_{0}\right)$ in which a continuous ULA will be obtained. The connection between $\overset{⌢}{p}\left(\omega_{h}\right)$, $\overset{⌢}{p}\left(\omega_{0}\right)$ with ${\overset{⌢}{p}}_{\mathrm{optimal}}\left(\omega_{0}\right)$ is defined as $f:\left\{\overset{⌢}{p}\left(\omega_{0}\right),\overset{⌢}{p}\left(\omega_{h}\right)\right\}\to {\overset{⌢}{p}}_{\mathrm{optimal}}\left(\omega_{0}\right)$ (h=1,2,⋯,H).

As the $\overset{⌢}{p}\left(\omega_{h}\right)$ and $\overset{⌢}{p}\left(\omega_{0}\right)$ represent the virtual sensors positions in ${\overset{⌢}{c}}_{\mathrm{vec}}$, the connection between ${\overset{⌢}{c}}_{\mathrm{vec}}\left(\omega_{0}\right)$, ${\left\{{\overset{⌢}{c}}_{\mathrm{vec}}\left(\omega_{h}\right)\right\}}_{h=1}^{H}$ and ${\overset{⌢}{c}}_{\mathrm{vec}\_\mathrm{optimal}}\left(\omega_{0}\right)$ is the same with f where ${\overset{⌢}{c}}_{\mathrm{vec}\_\mathrm{optimal}}\left(\omega_{0}\right)$ corresponds to the continuous 2*q*th-order difference co-array without holes. Therefore, ${\overset{⌢}{c}}_{\mathrm{vec}\_\mathrm{optimal}}\left(\omega_{0}\right)$ can be obtained by filling in the missing elements with ${\overset{⌢}{c}}_{\mathrm{vec}}\left(\omega_{0}\right)$, ${\left\{{\overset{⌢}{c}}_{\mathrm{vec}}\left(\omega_{h}\right)\right\}}_{h=1}^{H}$, according to f.

In fact, there is no need to calculate all the 2*q*th-order cumulant of the received signal in frequency $\omega_{h}$. Only the elements that are used to fill the holes are needed, which will relieve the computation burden and improve the efficiency of the method.

To explain the method more clearly, the four-level nested array in Figure 1 is taken as an example. There are holes in position 55, 56, 58, 59. To fill the hole in 55, the element in 57 should be used and the required frequency is $\omega_{1}=\frac{55}{57}\omega_{0}$. As the virtual element in 57 is the fourth-order difference co-array of physical sensors in positions [5, 8, 35], only physical sensors in [5, 8, 35] need to operate at $\omega_{1}$ to generate the necessary elements in $\overset{⌢}{p}\left(\omega_{1}\right)$. Additionally, physical sensors in [5, 8, 35] operating at $\omega_{2}=\frac{56}{57}\omega_{0}$ will fill the hole in 56. Similarly, physical sensors in [5, 35] should be used to operate at a multi-frequency $\omega_{3}=\frac{58}{57}\omega_{0}$ and $\omega_{4}=\frac{59}{57}\omega_{0}$ to fill the holes in 58, 59. By filling the holes, the necessary elements of ${\overset{⌢}{c}}_{\mathrm{vec}}\left(\omega_{1}\right)\sim {\overset{⌢}{c}}_{\mathrm{vec}}\left(\omega_{4}\right)$ are obtained.

By matching the elements in $\overset{⌢}{p}\left(\omega_{1}\right)\sim \overset{⌢}{p}\left(\omega_{4}\right)$, $\overset{⌢}{p}\left(\omega_{0}\right)$ with the desired manifold vector ${\overset{⌢}{p}}_{\mathrm{optimal}}\left(\omega_{0}\right)$, the connection map between them is obtained. The continuous segment will be extended from $[-54,-53,\cdots ,54]d_{0}$ to ${\overset{⌢}{p}}_{\mathrm{optimal}}\left(\omega_{0}\right)=[-70,-69,\cdots ,70]d_{0}$. At last, the elements in ${\overset{⌢}{c}}_{\mathrm{vec}}\left(\omega_{0}\right)$, ${\overset{⌢}{c}}_{\mathrm{vec}}\left(\omega_{1}\right)\sim {\overset{⌢}{c}}_{\mathrm{vec}}\left(\omega_{4}\right)$ are used to construct the desired ${\overset{⌢}{c}}_{\mathrm{vec}\_\mathrm{optimal}}\left(\omega_{0}\right)$, according to the map f. It can be found that the effective DOFs increase obviously. Correspondingly, the DOA estimation performance will be promoted.

The procedure of the MFMFS method can be summarized as Algorithm 1.

**Algorithm 1.** Summary of the Multi-Frequency Method for Minimum Frequency Separation (MFMFS).**Input**: Receive signal sequence ${\left\{y\left(t\right)\right\}}_{t=1}^{L}$ of the 2*q*-level nested array from *D* sources. **Output**: The optimized cumulant vector ${\overset{⌢}{c}}_{\mathrm{vec}\_\mathrm{optimal}}\left(\omega_{0}\right)$.
**Step** **1**Ascertain the numbers of physical sensors and their positions of each level nested array.**Step** **2**Calculate the *2q*th-order cumulants of the received signal and form the *2q*th-order difference co-array.**Step** **3**Analyze the detailed structure of the 2*q*th-order difference co-array and find the holes.**Step** **4**Ascertain the sensors that operate at multi-frequency and the required frequencies.**Step** **5**Calculate the support vector of sensors operating at multi-frequency and compare them with the desired support vector ${\overset{⌢}{p}}_{\mathrm{optimal}}\left(\omega_{0}\right)$ to construct the connection map *f*.**Step** **6**Use the elements in ${\overset{⌢}{c}}_{\mathrm{vec}}\left(\omega_{0}\right)$, ${\overset{⌢}{c}}_{\mathrm{vec}}\left(\omega_{1}\right)$ etc. to construct the desired ${\overset{⌢}{c}}_{\mathrm{vec}\_\mathrm{optimal}}\left(\omega_{0}\right)$, according to the map in Step 5.


In summary, Reference [29] applies the multi-frequency methods in the co-prime array to improve the DOA estimation performance. They consider the condition of a second order statistic for co-prime array. First, we expand the method in 2*q*-th order cumulant-based nested array. The relationship is different and we have given a detailed relationship map of the algorithm. However, with the increase of the sensors number, the first method will be more and more complicated and time consuming, which is not practical in the real applications. In the next section, we will propose a novel strategy to use multi-frequency from the aspect of optimizing the physical sensors, which aims to balance the maximum DOFs and the fastest array design.

### 3.2. The Multi-Frequency Method for a Minimum Number of Frequencies (MFMNF)

The MFMFS method is designed under the requirement that the frequency separation is the minimum. However, the number of required frequencies equals half of the number of holes. When the number of physical sensors increases, the holes will increase rapidly and the number of frequencies should increase correspondingly. It is required that the sources must have proportional spectra at all frequencies, which is shown below.

(18) $$\frac{\sigma_{k}^{2}\left(\omega_{h}\right)}{\sigma_{l}^{2}\left(\omega_{h}\right)}=\beta_{k,l},$$

where $\beta_{k,l}$ denotes the power ratio of source k and source l. It indicates that the power ratio of different frequencies should remain constant for all frequencies. Therefore, with the increase of the number of needed frequencies, the non-proportional spectra problem, which degrades the DOA estimation performance and will become more serious. Therefore, we propose another method that can fill all the holes with only one frequency.

Different from the above method which chooses the required frequencies based on the 2*q*th-order difference co-array, the MFMNF method chooses frequency by analyzing the original physical array. We find that by selecting the proper physical sensors, only one frequency is needed to fill all the holes, which satisfies the requirement of a minimum number of frequencies.

According to the specific requirements, there are two situations.

In the first situation, if the used sensors are required to be as few as possible, we can let the last $\left\lfloor N_{2q}/2\right\rfloor$ physical sensors work at a frequency of $\omega_{1}=\alpha \omega_{0}$, where $N_{2q}$ is the number of sensors of the 2*q*th level ULA and α is an integer. The problem is to find the best α, which can yield a continuous 2*q*th-order difference co-array with maximum DOFs. By numerous experiments, we can find that when α=2, the holes in the former 2*q*th-order difference co-array are filled and the continuous ULA has the maximum number of virtual sensors. Taking the 2*q*-level nested array in Figure 1 as an example, its fourth level ULA contains two sensors such as $N_{2q}=2$. If the last $\left\lfloor N_{2q}/2\right\rfloor =1$ sensor known as the physical sensor in 35, work at $2\omega_{0}$, then the continuous ULA on the positive side will extend to 89, which is larger than the MFMFS method. This method actually changes the structure of the former 2*q*th-order difference co-array and fills the holes in the difference co-array from a different aspect.

In the second situation, if there is no limit on the number of required physical sensors, the continuous ULA can be further increased. Let all the physical sensors work at frequency $\omega_{1}=\alpha \omega_{0}$ where α is an integer. The positions of virtual sensors in the 2*q*th-order difference co-array consist of three parts including the self-difference co-array $S_{0}$ and $S_{1}$ and the cross difference co-array $S_{c}$ and their expressions are shown below.

(19) $$S_{0}=\left\{\sum_{i=1}^{q}z_{i}-\sum_{k=q+1}^{2q}z_{k},\;z_{i},z_{k}\in \left\{x_{n},\;n=1,2,\cdots ,N\right\}\right\},$$

(20) $$S_{1}=\left\{\sum_{i=1}^{q}{z^{\prime }}_{i}-\sum_{k=q+1}^{2q}{z^{\prime }}_{k},\;{z^{\prime }}_{i},{z^{\prime }}_{k}\in \left\{x_{n},\;n=1,2,\cdots ,N\right\}\right\},$$

(21) $$S_{c}=\left\{\alpha \left(\sum_{i=1}^{q}{z^{\prime }}_{i}-\sum_{k=q+1}^{2q}{z^{\prime }}_{k}\right)-\left(\sum_{i=1}^{q}z_{i}-\sum_{k=q+1}^{2q}z_{k}\right)\right\}.$$

Therefore, the sensors positions of the 2*q*-level nested array working at two frequencies can be expressed as $S\left(\alpha \right)=S_{0}\cup S_{1}\cup S_{c}$. When applying the MUSIC, ESPRIT, or other subspace based method, we wish to maximize the number of virtual sensors in the continuous part of S(α). Set the DOFs of the continuous ULA of the 2*q*th-order difference co-array as $S_{\mathrm{con}}\left(\alpha \right)$ where $S_{\mathrm{con}}\left(\alpha \right)\subset S\left(\alpha \right)$. Then the problem turns to find the best α to maximize the $S_{\mathrm{con}}\left(\alpha \right)$, which is shown below.

(22) $$\underset{\alpha }{\arg}\;\max S_{\mathrm{con}}\left(\alpha \right).$$

However, since it is hard to obtain the closed form expression of $S_{\mathrm{con}}\left(\alpha \right)$, it is impossible to get the closed form expression of the optimal α. This problem can only be solved by searching through all the possible solutions and select the best α.

The DOA estimation performance has a close relationship with the DOFs, which is decided by the types of array and the used methods. For a comparison, we list the DOFs of different methods in Table 1. The commonly used random array and MRA are based on the second order statistic. Since the element positions of a random array are completely random, it does not have definitive DOFs. For comparison, we take one example for each number of physical sensors and the corresponding DOFs are listed in the second column of Table 1. The DOFs of the MRA are listed as the third column of Table 1. The last column gives the best additional frequency coefficients for the MFMNF2 method.

We can find that the DOFs of a random array and MRA are both far smaller than that of other arrays and their DOA estimation performance will be worse than other arrays in Table 1. As a result, there is no need to consider their performance in the simulations. For the MFMNF1 and MFMNF2 method, we can find that, by using more sensors, the DOFs will be further improved. Except for the increase of the DOFs, there is another advantage that the requirement of proportional spectra of sources is easier to satisfy. Therefore, the DOA estimation performance will be improved. It can be predicted that the DOFs of the continuous ULA segment will be further increased by introducing more frequencies into the array. However, the optimization problem will become more complicated. Furthermore, the requirement on the proportional spectra of sources will be harder to satisfy.

Generally speaking, except for *N* = 7, the DOFs of the MFMFS method is close or less than the OLA-MR in Reference [23]. The DOFs of MFMNF1 are bigger than the OLA-MR when *N* = 5, 6, 7, and less than the OLA-MR when *N* = 4 and 8. However, for the MFMNF2 method, the DOFs are far bigger than the OLA-MR. It is because the application of multi-frequency forms new virtual sensors and expands the array.

In a word, the MFMFS expanded the multi-frequency methods from the second order statistic case to the 2*q*th-order case and gives detailed procedures to fill the holes in the 2*q*th-order difference co-array. The MFMNF1 and MFMNF2 solve this problem from a novel aspect and can use only one frequency to fill the holes and expand the DOFs. That will avoid the iterative calculations of the 2*q*th-order cumulants and the non-proportional spectra problems brought by introducing many frequencies can also be solved. Therefore, the MFMNF1 and MFMNF2 are both efficient and easy to realize.

The procedure of the second method can be summarized as Algorithm 2.

**Algorithm 2.** Summary of the Multi-Frequency Method for a Minimum Number of Frequencies (MFMNF).**Input**: Receive signal sequence ${\left\{y\left(t\right)\right\}}_{t=1}^{L}$ of the 2*q*-level nested array from *D* sources **Output**: The optimized cumulant vector ${\overset{⌢}{c}}_{\mathrm{vec}\_\mathrm{optimal}}\left(\omega_{0}\right)$.
**Step** **1**Ascertain the numbers of physical sensors and their positions of each level nested array.**Step** **2**If the used sensors are required to be as few as possible, let the last $\left\lfloor N_{2q}/2\right\rfloor$ physical sensors work at $2\omega_{0}$ to form the needed continuous virtual ULA. If there is no limit on the number of required physical sensors, let all the physical sensors work at frequency $\omega_{1}=\alpha \omega_{0}$ to form the needed virtual array element. In the second case, a search through all possible α will be needed.**Step** **3**Calculate the *2q*th-order cumulants of the received signals and form the *2q*th-order difference co-array.**Step** **4**Construct the desired ${\overset{⌢}{c}}_{\mathrm{vec}\_\mathrm{optimal}}\left(\omega_{0}\right)$.


### 3.3. Spatial Smoothing Based Algorithm for the Multi-Frequency 2q-Level Nested Array

After filling the holes in the 2*q*th-order difference co-array, the signal model can be expressed by the equation below.

(23) $${\overset{⌢}{c}}_{\mathrm{vec}\_\mathrm{optimal}}\left(\omega_{0}\right)=A_{\mathrm{optimal}}\left(\theta \right)\eta \left(\omega_{0}\right),$$

where ${\overset{⌢}{c}}_{\mathrm{vec}\_\mathrm{optimal}}\left(\omega_{0}\right)\in {\mathbb{C}}^{\left(2N_{o}+1\right)\times 1}$ and $A_{\mathrm{optimal}}\left(\theta \right)\in {\mathbb{C}}^{\left(2N_{o}+1\right)\times D}$. $N_{o}$ is the number of continuous positive elements in the 2*q*th-order difference co-array. Equation (23) is equivalent to the received signal model of $\left(2N_{o}+1\right)$ element ULA. The 2*q*th-order cumulants of the Gaussian is zero when q≥2, so there is no noise term in Equation (23). However, because the one column source vector $\eta \left(\omega_{0}\right)$ behaves like correlated sources, it cannot be directly used to estimate the DOAs. The classical Spatial Smoothing (SS) technique [30,31] should be applied to solve this problem. Divide the ${\overset{⌢}{c}}_{\mathrm{vec}\_\mathrm{optimal}}\left(\omega_{0}\right)$ into sub-vectors ${\overset{⌢}{c}}_{\mathrm{sub}\_i}\left(\omega_{0}\right)\in {\mathbb{C}}^{\left(N_{o}+1\right)\times 1}$($i=1,2,\cdots ,N_{o}+1$) where ${\left[{\overset{⌢}{c}}_{\mathrm{sub}\_i}\left(\omega_{0}\right)\right]}_{m}$=${\left[{\overset{⌢}{c}}_{\mathrm{vec}\_\mathrm{optimal}}\left(\omega_{0}\right)\right]}_{i+m-1}$. Calculate the covariance matrix of each ${\overset{⌢}{c}}_{\mathrm{sub}\_i}\left(\omega_{0}\right)$ and their average covariance matrix as:

(24) $$C_{\mathrm{optimal}}=\frac{1}{N_{o}+1}\sum_{i=1}^{N_{o}+1}{\overset{⌢}{c}}_{\mathrm{sub}\_i}{\overset{⌢}{c}}_{\mathrm{sub}\_i}^{H},$$

At last, subspace based algorithms such as MUSIC or ESPRIT can be applied to estimate DOAs based on $C_{\mathrm{optimal}}$. It should be mentioned that, since the subspace-based algorithm is used for DOA estimation, the number of sources is assumed to be known.

### 3.4. Discussions on the Computational Complexity and Cramér–Rao Bound

#### 3.4.1. Computational Complexity

The main steps are calculated of 2*q*th-order cumulants, spatial smoothing, eigen value decomposition, and peaks finding. The computation complexity of these four steps are $O\left(LN^{2q}\right)$, $O\left(LN^{2q}\right)$, $O\left(N^{6q}\right)$, and $O\left(N^{2q}\left(N^{2q}-K\right)\frac{\Theta }{\Delta \theta }\right)$, respectively, where Θ is the range of angle and Δθ is the searching step. In summary, the computation complexity of the 2*q*th-order cumulant based MUSIC is $O\left(LN^{2q}+N^{6q}+N^{2q}\left(N^{2q}-K\right)\frac{\Theta }{\Delta \theta }\right)$. For the MFMFS method, the virtual array are $O\left(\frac{H}{2}LN^{2q}\right)$ where H is the number of holes. Therefore, its computation complexity is $O\left(\frac{H}{2}LN^{2q}+N^{6q}+N^{2q}\left(N^{2q}-K\right)\frac{\Theta }{\Delta \theta }\right)$. For the MFMNF1 method, one more sensor is used, which is N+1. For MFMNF2, the number of used sensors is actually 2N. We can find that, for complexity in terms of Landau’s big O notation, they are all in the same level. Therefore, these algorithms all have similar complexity including $O\left(LN^{2q}+N^{6q}+N^{2q}\left(N^{2q}-K\right)\frac{\Theta }{\Delta \theta }\right)$.

#### 3.4.2. Cramér–Rao Bound

Cramér–Rao Bound (CRB) gives the bound for DOA estimation performance. It can be deduced by calculating the inverse of the Fisher information matrix (FIM). As mentioned in Reference [20], there exist two kinds of situations including the deterministic CRB for the deterministic (nonrandom) unknown sources and the stochastic CRB for the random sources. The sources in this paper are assumed to be non-Gaussian random. The stochastic CRB should be studied. However, since the sources are assumed to be BPSK modulated and the 2*q*th-order cumulants are calculated, the closed form expression of the CRB is hard to obtain in this case and References [32,33] only give the cases when the source number is one or two. 

According to Reference [33] for the single source case, the CRB is given by the formula below.

(25) $$\mathrm{CRB}\left(\theta_{1}\right)=\frac{1}{P}\left(\frac{1}{\gamma_{1}}\frac{\sigma_{u}^{2}}{\sigma_{1}^{2}}\right)\left(\frac{1}{1-f\left(\rho \right)}\right),$$

where $\rho =N\sigma_{1}^{2}/\sigma_{u}^{2}$ and $f\left(\rho \right)\overset{\mathrm{def}}{=}\left(e^{-\rho }/\sqrt{2\pi }\right)\int_{-\infty }^{+\infty }\left(e^{-\zeta^{2}/2}/\cosh\left(\zeta \sqrt{2\rho }\right)\right)d\zeta$. $\sigma_{1}^{2}$ and $\sigma_{u}^{2}$ denote the power of the source signal and noise, respectively. $\gamma_{1}$ is the purely geometrical factor with $2{a^{\prime }}_{1}^{H}\Pi_{a_{1}}^{⊥}{a^{\prime }}_{1}$ where ${a^{\prime }}_{1}=da_{1}/d\theta_{1}$, $\Pi_{a_{1}}^{⊥}=I_{N}-a_{1}a_{1}^{H}/N$, and $a_{1}$ is the steering vector.

For the two source cases, the signal model is $y\left(t\right)=\sum_{d=1}^{2}a\left(\theta_{d}\right)s_{d}\left(t\right)+u\left(t\right)$ and the corresponding distribution turns to Reference [33].

(26) $$p\left(y\left(t\right)\right)=\frac{1}{L^{2}\pi^{N}\sigma_{u}^{2N}}\sum_{j=1}^{4}e^{-{\left\|y\left(t\right)-As_{j}\right\|}^{2}/\sigma_{u}^{2}},$$

where $s_{j}={\left(\sigma_{1}\varepsilon_{j,1}e^{i\phi_{1}},\sigma_{2}\varepsilon_{j,2}e^{i\phi_{2}}\right)}^{T}$ and $\left(\varepsilon_{j,1},\varepsilon_{j,2}\right)=\left(\pm 1,\pm 1\right)$. If we denote all the unknown variables in Equation (26) as $\psi ={\left(\sigma_{u},\sigma_{1},\phi_{1},\theta_{1},\sigma_{2},\phi_{2},\theta_{2}\right)}^{T}$ to obtain the FIM, we have to deduce the partial derivative of Equation (26), which is shown below.

(27) $$\frac{1}{P}{\left(F\right)}_{k,l}=\underset{P\to \infty }{\lim}\sum_{t=1}^{P}\left(\frac{\partial \ln p\left(y\left(t\right);\psi \right)}{\partial \psi_{k}}\right)\left(\frac{\partial \ln p\left(y\left(t\right);\psi \right)}{\partial \psi_{l}}\right),$$

where $\psi_{k}$ and $\psi_{l}$ represent the elements in ψ. The CRB can be yielded by calculating the inverse of the FIM. However, as $\psi ={\left(\sigma_{n},\sigma_{1},\phi_{1},\theta_{1},\sigma_{2},\phi_{2},\theta_{2}\right)}^{T}$ contains seven variables, the computation of the partial derivative will be quite complex. It can be inferred that, when the number of targets is larger especially when *D* >= *N*, the corresponding CRB is prohibitive to compute. Therefore, the performance guarantee is a complex problem and needs further research and discussion.

## 4. Simulation

In this section, several simulations are performed to study the DOA estimation performance of the proposed method. 

### 4.1. Simulation 1

**Figure 2:**
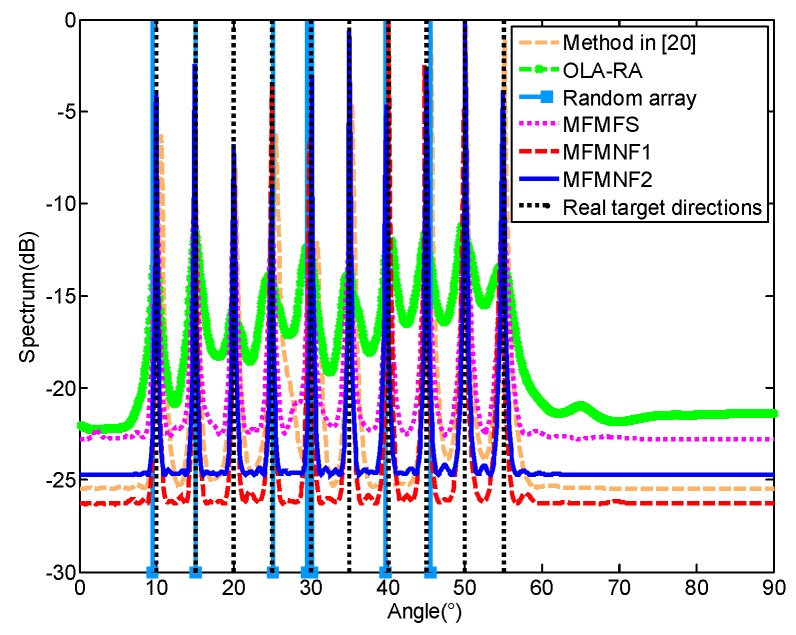
Spatial spectra of different methods.

First, we study the multi-targets estimation performance of the proposed methods. A four-level nested array with seven physical sensors, their positions, and the virtual fourth-order difference co-array is shown in Figure 1. *D* = 10 targets uniformly distribute from 10° to 55°. The SNR = 10 dB and the number of snapshots is 500. The spatial spectra of different methods are shown in Figure 2. The ‘OLA-MR’ denotes the optimal linear array with minimum redundancy, which also adopts the spatial smoothing MUSIC based on the single frequency 2*q*th-order cumulant as the method in Reference [20]. We set that the random array has seven sensors with sensor positions [0, 5, 10, 18, 23, 29, 40]. The random array adopts the MBMP algorithm in Reference [7] to recover the directions of targets and the number of nodes of each level is set as [3, 2, 1, 1, 1, 1, 1, 1, 1, 1]. For simplicity, the MFMNF1 represents the first case that the used physical sensors are required to be as few as possible and the MFMNF2 represents the case that all physical sensors can be used. The MFMFS, MFMNF1, and MFMNF2 all adopt spatial smoothing MUSIC based on the multi-frequency method. It can be found that only the OLA-MR, MFMFS, MFMNF1, and MFMNF2 can accurately estimate all the directions of 10 targets. As shown in Figure 2, The estimation result of the random array is [9.5°, 15.1°, 25.1°, 29.5°, 29.7°, 29.9°, 30.1°, 30.2°, 39.7°, 45.5°]. However, it fails to estimate the directions of the 10 targets. This matches with the simulation result in Reference [7]. The Figure 2 in Reference [7] demonstrates that, for a phased array when the *N* = 11, *D* = 5, the probability of the DOA recovery error is almost 1, which means the array with 11 sensors cannot find the five targets accurately while the array with seven sensors estimate 10 targets.

Next, we further study the RMSE versus SNR and the number of snapshots of the proposed methods. The number of targets and their directions are the same with the above simulation. A total of 500 trials are taken to examine the performance. The RMSE is defined by the equation below.

(28) $$\mathrm{RMSE}={\left[\frac{1}{500D}\sum_{d=1}^{D}\sum_{r=1}^{500}{\left({\hat{\theta }}_{d}^{r}-\theta_{d}\right)}^{2}\right]}^{1/2},$$

where $\theta_{d}$ is the real direction of the *d*th target and ${\hat{\theta }}_{d}^{r}$ is the estimated angle of the *d*th target in the *r*th trial.

**Figure 3:**
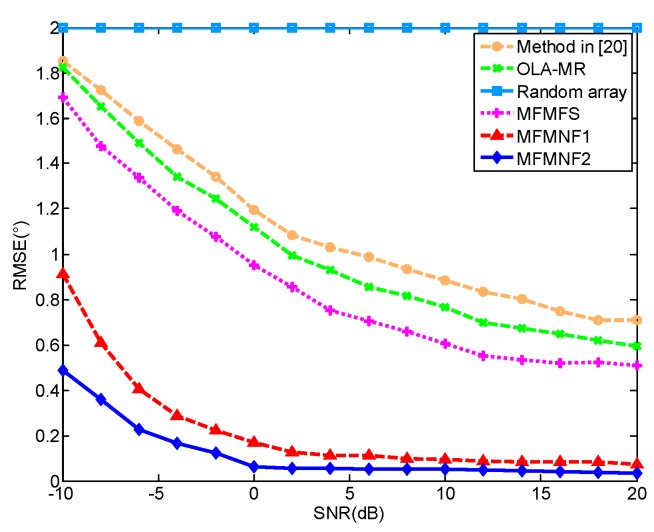
RMSEs versus SNR.

Figure 3 shows the RMSEs versus SNR of these methods. In this case, the number of snapshots is 500. Other than the random array that fails to estimate the DOAs of these targets, the RMSEs of other methods all get smaller with the increase of SNR. Among them, the MFMNF2 has the best performance and the MFMNF1 is the sub-optimal method.

**Figure 4:**
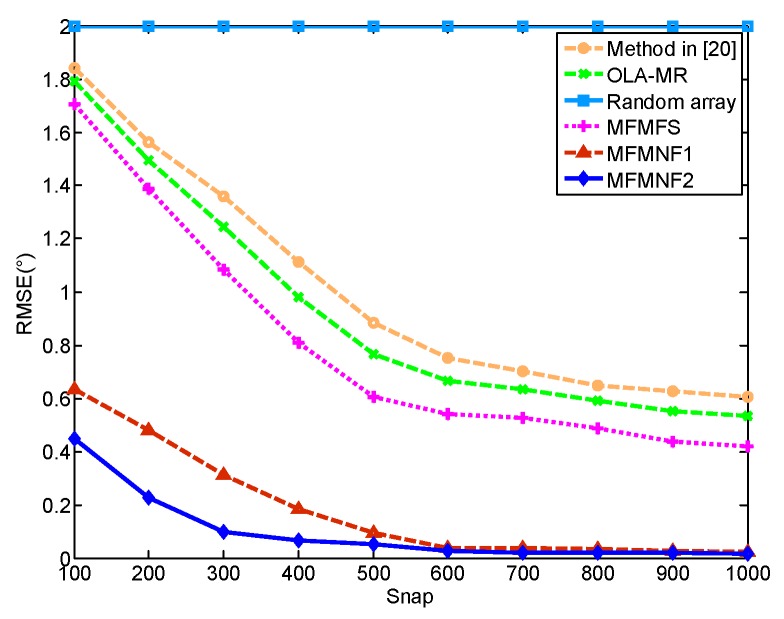
RMSEs versus the number of snapshots.

Similarly, Figure 4 shows the RMSE versus the number of snapshots for these methods where SNR = 10 dB. Except for the random array that fails to estimate the DOAs of these targets, the RMSEs of other methods all get smaller with the increase of the number of snapshots. The MFMNF1 has the best performance and the MFMFS performs worse than MFMNF1 and MFMNF2 but better than the OLA-MR method and the method in Reference [20].

### 4.2. Simulation 2

**Figure 5:**
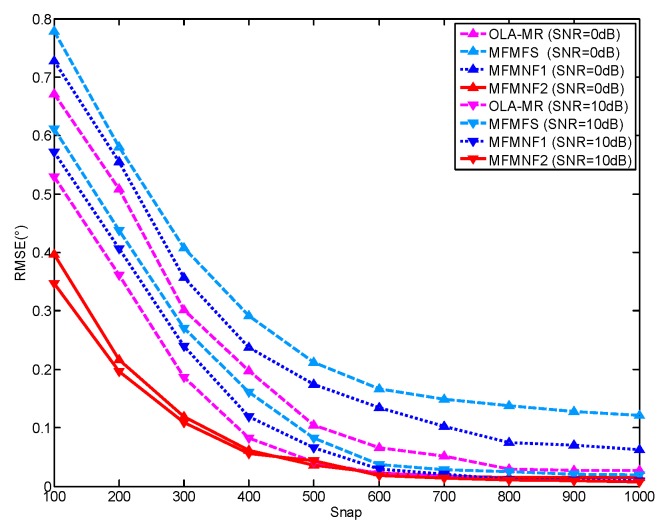
RMSE under a varying number of snapshots for different SNR values.

To further analyze the DOA estimation performance, we conduct a simulation pertaining to a varying number of snapshot samples for different SNR values (SNR = 0 dB or 10 dB) in Figure 5. Since Figure 3 has shown that the random array is not applicable to overestimate the situation, we mainly compare the DOA estimation performance of the OLA-MR with the methods proposed in this paper. The number of physical sensors is *N* = 8. The sensor positions of the OLA-MR are [0, 1, 2, 3, 51, 58, 72, 84]. For a better comparison, the setting of the targets is the same with Simulation 1 including 10 targets from directions $\left[10^{\circ }:5^{\circ }:55^{\circ }\right]$. It can be found that the DOA estimation is more accurate when SNR is higher. Since the OLA-MR has more effective DOFs when *N* = 8, it performs better than MFMFS and MFMNF1. However, the difference is within 0.2°. For methods in the same SNR, the MFMNF2 has the best performance. Therefore, the methods proposed in this paper have better or close DOA estimation performance. The methods in this paper do not need iterative optimization and are easy to realize. Therefore, the proposed methods are more comparatively practical.

### 4.3. Simulation 3

**Figure 6:**
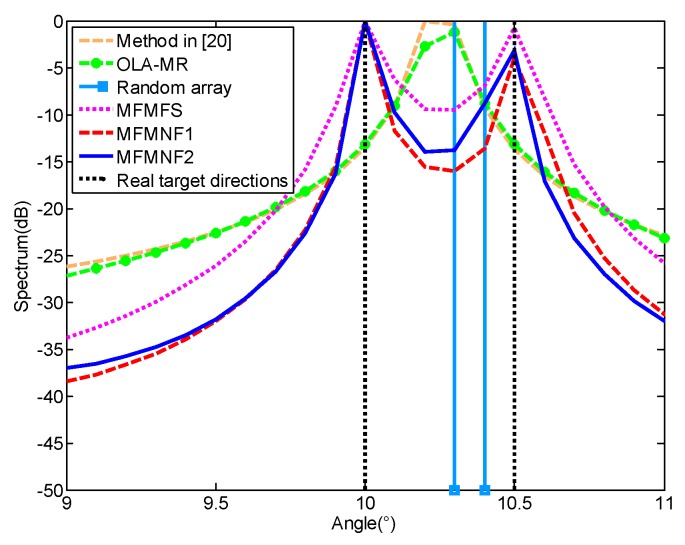
MUSIC spectra for *D* = 2 sources on a ULA with seven sensors.

In this simulation, we consider two closely spaced targets with angles [10°, 10.5°]. The number of snapshots is 300 and SNR = 10 dB. Other parameter settings are the same with Simulation 1. The spatial spectra for different methods are presented in Figure 6. It can be found that only the proposed MFMFS, MFMNF1, and MFMNF2 methods can accurately distinguish the two targets. The random array can distinguish between the two targets but there exist some estimation errors. In addition, the method in Reference [20] and the OLA-MR method both fail to distinguish between the two closely spaced targets.

**Figure 7:**
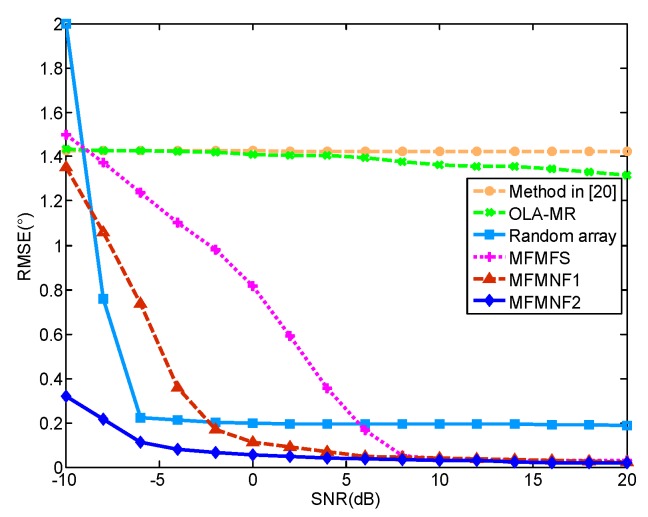
RMSEs versus SNR.

Figure 7 shows the RMSEs versus SNR of these methods. In this case, the number of snapshots is 500. With the increase of SNR, the RMSEs of the random array including the MFMFS, MFMNF1, and MFMNF2 methods all get smaller and the MFMNF2 has the best performance. When SNR > 5 dB, the RMSE of a random array remains around 0.2°. The OLA-MR method and the method in Reference [20] fail to distinguish between the two targets.

**Figure 8:**
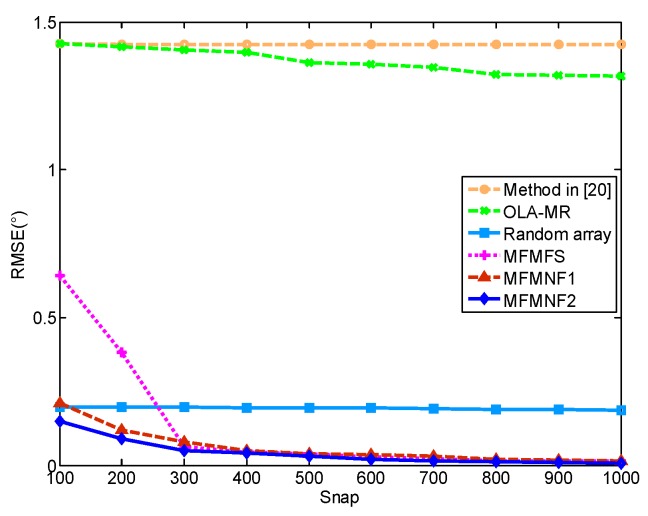
RMSEs versus SNR.

Similarly, Figure 8 shows the RMSE versus the number of snapshots for these methods where SNR = 10 dB. The RMSEs of the MFMFS, MFMNF1, and MFMNF2 methods all get smaller with the increase of a number of snapshots and the MFMNF2 has the best performance. The RMSE of a random array remains around 0.2°. The MFMFS performs worse than MFMNF1 and MFMNF2 but better than the methods before. The OLA-MR and the method in Reference [20] fail to distinguish between the two targets.

## 5. Conclusions

In this paper, we propose two multi-frequency methods, which are suitable for different restrictions in a 2*q*-level nested array to improve the DOA estimation performance. The combination of 2*q*th-order cumulant and the 2*q*th-order difference co-array results in the expanded DOFs. The 2*q*-level nested array has definite expression for sensor positions and their 2*q*th-order difference co-array will contain a ULA with $O\left(N^{2q}\right)$ virtual sensors. However, there exist holes between the continuous segment and the discrete segment. The application of multi-frequency fills the holes and expands the effective DOFs. The DOA estimation performance is, therefore, improved. The simulations validate the performance of the proposed method. More importantly, the second order difference co-array of nested array has no holes. However, when q≥2, holes appear in the 2*q*th-order difference co-array. This indicates that the 2*q*th-order cumulants lead to new characteristics of the array and is worth further research. Therefore, the multi-frequency methods will have a wide application in the related research.

## Figures and Tables

**Table 1:** DOFs of different methods.

Number of Physical Sensors	Random Array	MRA	2*q*-Level Nested Array	OLA-MR in [23]	MFMFS	MFMNF1	MFMNF2	Best α
4	9	11	29	49	29	45	133	8
5	17	19	49	87	61	93	241	12
6	25	27	73	93	93	119	419	8
7	31	35	109	123	141	179	597	32
8	39	47	163	289	213	269	1049	18
